# Workplace violence in Mato Grosso do Sul, Brazil (2012-2022): temporal trends and epidemiological analysis

**DOI:** 10.47626/1679-4435-2026-1535

**Published:** 2026-02-27

**Authors:** Alberth Rangel Alves de Brito, Ilias de Musis, Ediney José da Silva Magalhães, Eber Lucas Fernandes Saucedo, Andréia Insabralde de Queiroz Cardoso, Luciana Contrera

**Affiliations:** 1 Universidade Federal de Mato Grosso do Sul, Instituto Integrado de Saúde, Programa de Pós-Graduação Stricto Sensu em Enfermagem, Campo Grande, MS, Brazil; 2 Universidade Federal de Mato Grosso, Instituto de Ciências Exatas e da Terra, Laboratório de Estatística, Cuiabá, MT, Brazil

**Keywords:** violence, workplace violence, health information systems, temporal distribution., violência, violência no trabalho, sistemas de informação em saúde, distribuição temporal.

## Abstract

**Introduction:**

Workplace violence is highly prevalent in Brazil and worldwide, making it a priority on the public health agenda. This condition hinders the performance of work activities and contributes to the physical and mental illness of workers.

**Objectives:**

To analyze the temporal trend of notifications of workplace violence in the state of Mato Grosso do Sul, Brazil.

**Methods:**

This was a quantitative time-series study using secondary data. A total of 699 notifications between 2012 and 2022 were analyzed using descriptive statistics and Prais-Winsten regression trend analysis.

**Results:**

Most victims were female (53.6%), of mixed race/skin color (38.6%), and aged between 25 and 45 years (51.1%). Physical violence was present in most notifications (68%), mainly through physical force/beating (53.5%). The majority of perpetrators were male (66.5%), and most acts of violence were committed by a single perpetrator (65.4%).

**Conclusions:**

There is a need for public policies aimed at workplace violence in order to ensure a safe work environment, as well as to promote the prevention, protection, and recovery of workers.

## Resumo

**Introdução:**

A violência relacionada ao trabalho apresenta elevada prevalência no Brasil e no mundo, configurando-se como uma das prioridades na agenda da saúde pública. Esse agravo prejudica o desenvolvimento das atividades laborais e contribui para o adoecimento físico e mental dos trabalhadores.

**Objetivos:**

Analisar a tendência temporal das notificações de violência relacionada ao trabalho em Mato Grosso do Sul, Brasil.

**Métodos:**

Estudo de série temporal, quantitativo, realizado a partir de dados secundários. Foram analisados 699 registros referentes ao período de 2012 a 2022, por meio de estatística descritiva e análise de tendência por regressão de Prais-Winsten.

**Resultados:**

As principais vítimas foram pessoas do sexo feminino (53,6%), de raça/cor de pele parda (38,6%) e com idade entre 25 e 45 anos (51,1%). Verificou-se que a violência física esteve presente na maioria das notificações (68%), principalmente agressão por meio de força corporal/espancamento (53,5%). Em relação aos autores da violência, evidenciou-se que, em sua maioria, eram do sexo masculino (66,5%) e que os atos foram praticados por um único autor (65,4%).

**Conclusões:**

Verificou-se a necessidade de políticas públicas direcionadas à violência relacionada ao trabalho, a fim de garantir ambientes laborais seguros, bem como fomentar a prevenção, proteção e recuperação dos trabalhadores.

## INTRODUCTION

According to the Occupational Safety and Health Administration, workplace violence refers to any act or threat of physical violence, harassment, intimidation, or other threatening behavior capable of causing harm, distress, or fear to workers. It ranges from threats and verbal abuse to physical assault and even homicide [1].

Violence experienced in the occupational setting leads to demotivation and dissatisfaction among workers, negatively affecting job performance and contributing to the development of work-related illness [2]. In addition, the consequences of this type of violence are often considered traumatic, making victims susceptible to health problems such as physical and psychological trauma [3].

Workplace violence is considered a complex phenomenon because it involves not only the identification of perpetrators and victims, but also the classification of its different types, as well as appropriate referral for health care and treatment [4]. The extent of its impact, particularly with regard to psychological harm, is also difficult to ass, since victims often remain silent in response to aggression due to feelings of shame, humiliation, or fear of retaliation, especially in the workplace [5].

The consequences and effects on the health and well-being of individuals who experience workplace violence are wide-ranging and may extend into the social domain, manifesting as physical and emotional exhaustion [4]. In this context, social and emotional withdrawal, social conflicts, and the reproduction of violence are common consequences. In this sense, experiences in the workplace play a significant role in shaping individual behavior within broader social contexts [6].

Workplace violence also has repercussions at the institutional level. The suffering experienced by workers significantly affects work performance and the performance of tasks and hurts interpersonal relationships. To illustrate the magnitude of this issue, when a worker is subjected to violence and others witness or are indirectly exposed to the event, such incident may disrupt work dynamics at both the individual and organizational levels [7].

Given the magnitude of the problem, studies have reported high rates of violence against professionals in the health, education, and public security sectors [8,9]. Nevertheless, despite evidence highlighting the vulnerability of certain occupational groups, research focusing on Brazilian workers remains limited. Therefore, the present study aimed to conduct an epidemiological analysis and assess temporal trends in notifications of workplace violence in Mato Grosso do Sul, Brazil.

## METHODS

### STUDY DESIGN AND PERIOD

This quantitative time-series study used secondary data to estimate the annual percentage change (APC) in workplace violence in Mato Grosso do Sul between January 1, 2012, and December 31, 2022, and to describe the epidemiological profile and outcomes of reported cases.

### DATA SOURCE

Notifications recorded in the Notifiable Diseases Information System (*Sistema de Informação de Agravos de Notificação* - SINAN) were analyzed. The data were collected in June 2023, following approval of the study by the Mato Grosso do Sul State Department of Health and the local research ethics committee. After receiving access to the database, notifications containing the response “yes” in the field “workplace violence” were identified and included in the analysis.

### STUDY VARIABLES

The variables analyzed included sociodemographic characteristics, such as year of notification, sex, race/skin color, age group, educational level, and macroregion of residence. Information on occupational groups, classified according to the Brazilian Classification of Occupations (*Classificação Brasileira de Ocupações*), and the issuance of a Workplace Incident Report were also considered. Variables related to the study outcomes included recurrence of violence, number of individuals involved, sex of the suspected perpetrator, type of violence, and means of aggression. Importantly, the variables “type of violence” and “means of aggression” include an “other” category, allowing for a detailed description of the event, since the options available on the interpersonal/self-inflicted violence notification form may not fully reflect the victim’s account of the violent episode.

Ages recorded in the notification forms were categorized into four groups, as the database allows age to be recorded in hours, days, months, or years, and in some cases only the date of birth is provided. Accordingly, ages were classified as < 25 years, 25 to < 45 years, ≥ 45 years, and not reported. Educational level was categorized in terms of years of schooling, given that this variable includes multiple response options, allowing the notifier to select the most appropriate category.

Occupational groups were classified according to the Brazilian Classification of Occupations, allowing a general description of the professions most affected by workplace violence. It should be noted that the notification forms, and consequently the information made available through SINAN, allow for the occupation of the individual to be either reported or left unspecified.

### STUDY SETTING

The macroregions of Mato Grosso do Sul were defined according to the Mato Grosso do Sul State Health Plan 2020-2023, issued by the State Department of Health. Four macroregions were identified - Campo Grande, Dourados, Corumbá, and Três Lagoas -, with an additional category for notifications recorded within the state but referring to events that occurred outside the state.

### SAMPLE PROFILE CHARACTERIZATION

For the characterization of the sample profile, records containing information on occupation, workplace violence, or the issuance of a Workplace Incident Report were considered eligible. Notifications that did not meet these criteria were excluded from the analysis. As a result, 112,472 notifications were excluded and 699 were included in the study. It should be noted that the SINAN notification form also includes other types of violence not related to work.

### STATISTICAL ANALYSIS

Data were organized in Microsoft Excel® spreadsheets and exported to R software, version 4.3.2, for statistical analysis. Temporal trends were assessed using the Prais-Winsten method for generalized linear regression, with a 95% confidence interval (95%CI). The year of notification (2012-2022) was defined as the independent variable (x), while variables related to sociodemographic characteristics and violence outcomes, such as sex, age group, race/skin color, and type of violence, were considered dependent variables (y). Descriptive analyses were performed for the remaining study variables, including the development of maps to spatially represent the issuance of the Workplace Incident Report. The maps displayed the number of notifications by municipality, organized into quantile-based intervals and adjusted to improve data visualization.

APC estimates were considered statistically significant when the p-value was < 0.05. Positive APC values indicated an increasing trend, whereas negative values indicated a decreasing trend. When the p-value was > 0.05, the trend was considered stable, meaning that the time series exhibited stationary behavior.

### ETHICAL CONSIDERATIONS

This study was conducted in accordance with the ethical principles established by Resolution No. 466 of the Brazilian National Health Council, from December 12, 2012, and was approved by the Research Ethics Committee of the Federal University of Mato Grosso do Sul. Given the nature of the study, restricted-access secondary data were used, with no participant identification; records were anonymized and identified solely by unique, randomly assigned codes.

## RESULTS

From 2012 to 2022, a total of 699 workers were reported as having experienced workplace violence. Analysis of sociodemographic variables (Table 1) showed that 2022 recorded the highest number of notifications during the period, with 94 cases. Female workers predominated among the notifications, with this pattern remaining relatively stable from 2012 to 2021, except for a reversal of the trend observed in 2015 and again in 2022.

**Table 1:** Annual distribution of reported workplace violence cases according to sociodemographic variables and type of violence. Mato Grosso do Sul, 2012-2022 (number of cases = 699)

Variable	2012 Cases (%)	2013 Cases (%)	2014 Cases (%)	2015 Cases (%)	2016 Cases (%)	2017 Cases (%)	2018 Cases (%)	2019 Cases (%)	2020 Cases (%)	2021 Cases (%)	2022 Cases (%)	Total Cases (%)
Total	70	77	84	43	53	64	67	56	37	54	94	699
Sex												
Female	42 (60.0)	44 (57.1)	47 (56.0)	17 (39.5)	31 (58.5)	34 (53.1)	42 (62.7)	34 (60.7)	21 (56.8)	31 (57.4)	32 (34.0)	375 (53.6)
Male	28 (40.0)	33 (42.9)	37 (44.0)	26 (60.5)	22 (41.5)	30 (46.9)	25 (37.3)	22 (39.3)	16 (43.2)	23 (42.6)	62 (66.0)	324 (46.4)
Age group (years)												
< 25	23 (32.9)	26 (33.8)	26 (31.0)	9 (20.9)	11 (20.8)	11 (17.2)	22 (32.8)	12 (21.4)	10 (27.0)	10 (18.5)	23 (24.5)	183 (25.5)
25 to < 45	3 (44.3)	37 (48.1)	34 (40.5)	25 (58.1)	30 (56.6)	39 (60.9)	26 (38.8)	30 (53.6)	22 (59.4)	30 (55.5)	53 (56.4)	357 (52.0)
≥ 45	16 (22.9)	13 (16.9)	23 (27.4)	9 (20.9)	12 (22.6)	14 (21.9)	18 (26.9)	14 (25.0)	5 (13.5)	14 (25.9)	18 (19.1)	156 (22.1)
Not informed	0 (0.0)	1 (1.3)	1 (1.2)	0 (0.0)	0 (0.0)	0 (0.0)	1 (1.5)	0 (0.0)	0 (0.0)	0 (0.0)	0 (0.0)	3 (0.4)
Race/skin color												
White	27 (38.6)	23 (29.9)	36 (42.9)	18 (41.9)	20 (37.7)	27 (42.2)	25 (37.3)	20 (35.7)	18 (48.6)	18 (33.3)	37 (39.4)	269 (38.5)
Black	7 (10.0)	9 (11.7)	5 (6.0)	0 (0.0)	3 (5.7)	3 (4.7)	1 (1.5)	3 (5.4)	3 (8.1)	5 (9.3)	3 (3.2)	42 (6.0)
Mixed-race	25 (35.7)	26 (33.8)	30 (35.7)	18 (41.9)	17 (32.1)	25 (39.1)	28 (41.8)	28 (50.0)	12 (32.4)	23 (42.6)	38 (40.4)	270 (38.6)
Red (native Brazilian)	3 (4.3)	9 (11.7)	10 (11.9)	1 (2.3)	3 (5.7)	6 (9.4)	8 (11.9)	2 (3.6)	2 (5.4)	5 (9.3)	11 (11.7)	60 (8.6)
Asian descent	0 (0.0)	1 (1.3)	1 (1.2)	0 (0.0)	0 (0.0)	3 (4.7)	1 (1.5)	2 (3.6)	2 (5.4)	0 (0.0)	1 (1.1)	11 (1.6)
Not informed	8 (11.4)	9 (11.7)	2 (2.4)	6 (14.0)	10 (18.9)	0 (0.0)	4 (6.0)	1 (1.8)	0 (0.0)	3 (5.6)	4 (4.3)	47 (6.7)
Type of violence												
Physical violence	56 (80.0)	66 (85.7)	70 (83.3)	37 (86.0)	41 (77.4)	48 (75.0)	44 (65.7)	39 (69.6)	22 (59.5)	38 (70.4)	39 (41.5)	500 (72.2)
Sexual violence	2 (2.9)	0 (0.0)	1 (1.2)	0 (0.0)	3 (5.7)	3 (4.7)	4 (6.0)	2 (3.6)	5 (13.5)	0 (0.0)	3 (3.2)	23 (3.7)
Psychological/moral violence	5 (7.1)	7 (9.1)	2 (2.4)	2 (4.7)	1 (1.9)	9 (14.1)	6 (9.0)	2 (3.6)	2 (5.4)	2 (3.7)	40 (42.6)	78 (9.4)
Other	7 (10.0)	4 (5.2)	11 (13.1)	4 (9.3)	8 (15.1)	4 (6.3)	13 (19.4)	13 (23.2)	8 (21.6)	14 (25.9)	12 (12.8)	98

Source: Notifiable Diseases Information System (SINAN).

The age group with the lowest representation was workers aged 45 years or older. Regarding race/skin color, most notifications were concentrated among individuals who self-identified as White or mixed race/skin color, with the highest number of notifications recorded in 2022. The most prevalent type of violence was physical violence, with 500 reported cases, followed by psychological/moral violence, which also showed its highest number of notifications in 2022.

APC analysis of notifications across the four macroregions of Mato Grosso do Sul revealed a declining trend in the number of violence-related cases in the Campo Grande macroregion, with an average reduction of 4% over the study period. Decreasing trends were also observed among individuals under 25 years of age (APC = -8) and those aged 45 years or older (APC = -7.5), as well as among individuals of White race/skin color (APC = -67) and those with race/skin color not reported (APC = -83). A declining trend was likewise identified for cases classified as physical violence (APC = -7.3). Only the “other” category showed an increasing trend over the years (Table 2).

**Table 2:** Trends in the notification of workplace violence in the Campo Grande macroregion. Mato Grosso do Sul, 2012-2022 (number of cases = 699)

Variable	APC	95%CI	p-value	Trend
Total	-4.2	-7.5 to -0.8	0.023	Decreasing
Sex				
Female	-4	-9.5 to 1.9	0.160	Stable
Male	-4.2	-9.3 to 1.2	0.110	Stable
Age group (years)				
Less than 25	-8	-12 to -3.7	0.002	Decreasing
25 to under 45	-0.9	-5.1 to 3.4	0.650	Stable
45 years or more	-7.5	-13 to -1.4	0.023	Decreasing
Not informed	-13	-32 to 11	0.230	Stable
Race/skin color				
White	-67	-91 to 24	0.095	Decreasing
Indigenous Brazilian	-15	-45 to 31	0.430	Stable
Mixed-race	39	-59 to 373	0.560	Stable
Black	-55	-86 to 48	0.170	Stable
Asian descent	20	-15 to 68	0.270	Stable
Not informed	-83	-92 to -65	<0.001	Decreasing
Type of violence				
Physical violence	-7.3	-12 to -2	0.014	Decreasing
Psychological/moral violence	-1.4	-14 to 12	0.810	Stable
Sexual violence	12	-26 to 69	0.570	Stable
Other	7.3	2.7 to 12	0.005	Increasing

APC = annual percentage change.

Regarding the Três Lagoas macroregion, the number of notifications showed no significant variation between 2012 and 2022. However, declining trends were observed for the age group under 25 years (APC = -32) and for cases with missing information on race/skin color (APC = -27). The Dourados macroregion likewise showed no overall variation in the number of notifications. Nonetheless, increasing trends were identified among individuals aged 25 to under 45 years (APC = 4.3), in cases with missing information on race/skin color (APC = 66), and in cases classified as “other” (APC = 100). In contrast, physical violence exhibited a decreasing trend in this macroregion (APC = -4.9). The Corumbá macroregion showed no variation in the number of notifications over the study period. The issuance of Workplace Incident Reports for workplace violence varied across macroregions, with the highest number recorded in Campo Grande (n = 386), followed by Dourados (n = 225), Corumbá (n = 54), and Três Lagoas (n = 28).

**Figure 1:**
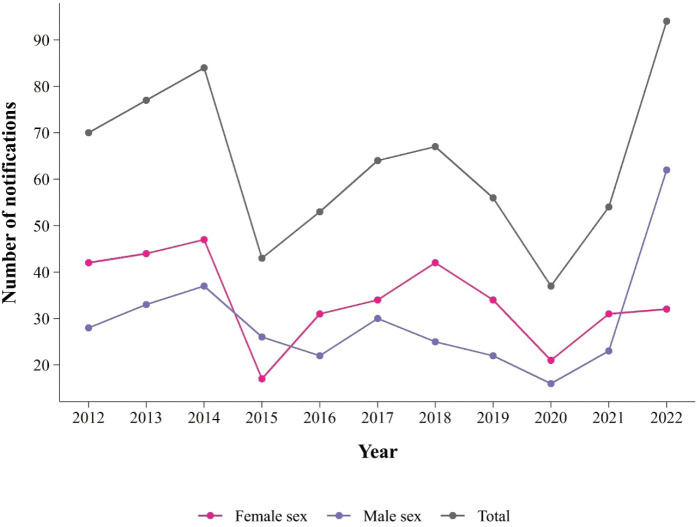
Distribution of workplace violence cases by sex. Mato Grosso do Sul, 2012-2022 (number of cases = 699).

Source: Notifiable Diseases Information System (SINAN).

Regarding the sociodemographic profile of victims, a higher frequency of workplace violence was observed among women (53.6%), except in 2015 and 2022, when a proportional increase in notifications among men was noted. In addition, most cases were perpetrated by a single individual (65.4%), predominantly male (66.5%). The most common means of aggression was physical force/beating (53.3%). Of note, an upward trend in notifications was observed between 2020 and 2022 for both sexes, with men accounting for the highest number of notifications during this period (Figure 1).

Analysis of information on the occurrence and number of individuals involved in workplace violence showed that 55.8% of cases represented first-time events and were most often perpetrated by a single individual (65.4%), predominantly male (66.5%). Physical force/beating was the most frequently reported means of aggression (53.5%). The Campo Grande macroregion accounted for the largest proportion of notifications (51.4%).

With regard to the sociodemographic characteristics of the victims, analysis of occupational groups showed that the category “not reported/ignored” was the most frequent (49.2%). This was followed by service and commerce workers (22.5%), agricultural, forestry, and fishing workers (6.9%), not elsewhere classified occupations such as students and retirees/pensioners (6.7%), professionals in science and the arts (5.3%), and administrative services workers (1.9%). All other occupational categories accounted for 1% or less of the notifications.

Consistent with the findings for occupational group, the variable “educational level” (categorized by years of schooling) also showed a high proportion of notifications classified as “not reported/ignored” (31.5%). This was followed by individuals with 1 to 9 years of schooling (29%), 10 to 12 years schooling (25.5%), and 13 years of schooling or more (14%).

## DISCUSSION

The present study identified epidemiological patterns in notifications of workplace violence recorded in SINAN. The analysis highlighted variations according to sex, race/skin color, age group, and macroregion of residence, as well as event-related characteristics, including place of occurrence, recurrence, number of individuals involved, sex of the suspected perpetrator, type of violence, and means of aggression.

Workplace violence was more prevalent among women, those self-identified as White or mixed race/skin color, individuals aged between 25 and < 45 years, and residents of the Campo Grande macroregion. Most notifications originated in the Campo Grande macroregion and involved a single aggressor, who was predominantly male. Physical violence was the most frequently reported type, with physical force/beating as the predominant means of aggression.

The predominance of female victims is consistent with findings from previous studies highlighting women’s vulnerability to workplace violence. Violence against women has become an increasingly visible social phenomenon, as reflected in media coverage, governmental reports, and academic discourse [10]. Patterns of workplace violence against women mirror their lived experiences and social roles within broader social contexts [11]. Nevertheless, persistent prejudice remains toward professional activities performed by women, which tend to be less respected than those carried out by men [12].

The variable “race/skin color” also warrants attention, as workplace violence may disproportionately affect different racial groups. Evidence suggests that individuals from certain ethnic backgrounds may be more frequently exposed to situations of moral harassment and discrimination, underscoring the importance of including this variable in analyses of the phenomenon [13]. These finding reinforce the need to understand how workplace violence affects individuals differently according to their racial characteristics.

Another relevant factor is the relationship between age and workplace violence. Although younger workers may be more vulnerable due to limited experience and lower job stability, data from the present study showed a higher number of notifications among adults aged 25 to < 45 years. A Brazilian study suggests that younger individuals may be more inclined to seek help when they perceive that parents or guardians are interested in understanding their experiences [14]. Moreover, younger workers are more likely to be exposed to workplace violence due to the nature of the jobs they occupy, their developmental stage, and their lack of prior experience compared with adult workers [15]. Thus, the lower prevalence of workplace violence notifications among younger individuals may result from insufficient parental or guardian support, as well as victims’ insecurity regarding the reporting process.

With regard to educational level, measured in years of schooling, similar proportions were observed between individuals classified as not reported or ignored (31.5%) and those who reported having 1 to 9 years of schooling (29%). A study conducted in Brazil showed that educational level is a determining factor in violence-related data and serves as a predictor of the occurrence of workplace violence. Thus, lower educational levels are associated with a higher likelihood of conflicts or workplace violence [16].

A Workplace Incident Report was issued in fewer than half of the cases analyzed. This document allows the verification of temporary work leave among individuals insured by the Brazilian National Social Security Institute [17]. The absence of this report in the remaining notifications may be attributed to the fact that SINAN allows the recording of different violence-related situations, including those involving individuals without formal employment contracts, such as students, retirees, and pensioners.

From a temporal perspective, an increase in notifications of workplace violence was observed between 2020 and 2022, possibly associated with the SARS-CoV-2 pandemic during the same period. Several studies have reported the occurrence of workplace violence among health care professionals [18]. In the present study, incomplete information was identified in a portion of the notifications, with the occupational category “ignored/not reported” accounting for the largest proportion (49.2%).

The reporting of workplace violence incidents in SINAN plays a key role in strengthening public health policies. Information on the location or setting of the violent event is essential for understanding its context, as well as for monitoring and surveillance activities [19]. Previous studies have documented a high number of health care visits by victims of workplace violence in family health units, outpatient clinics, and specialized centers, particularly for incidents occurring in residences and associated with domestic work [20,21].

In the analysis of regions of occurrence, the epidemiological health profile and the regional division of Mato Grosso do Sul were taken into account. According to the State Health Plan (2020-2023), the Campo Grande macroregion - comprising 34 municipalities across 11 microregions - has the largest population in the state, with 1,502,351 inhabitants [22]. This macroregion also showed the highest proportion of workplace violence cases (51.4%) compared with the others.

Regarding violence outcomes, a predominance of a single perpetrator per event was observed, who was typically male. The predominantly male authorship of aggressive acts is consistent with findings from previous studies and highlights the persistence of violent behavioral patterns [23]. Established research further indicates that male perpetrators of violence continue to represent a challenge for society, as male socialization often reinforces the resolution of conflicts through violent means [24].

In this study, cases of violence were classified according to their type and means of aggression. Physical violence, primarily involving physical force/beating, was present in more than half of the notified and analyzed cases. The occurrence of psychological or moral violence, as well as threats, was also noteworthy. The effects of workplace violence may lead to physical harm, strain social and family relationships, and contribute to psychological suffering, particularly in contexts marked by insecurity, hostility, and coercion [25].

## CONCLUSIONS

This study was instrumental in identifying the epidemiological profile of workplace violence cases reported in SINAN in Mato Grosso do Sul. Violent acts were predominantly physical in nature, most often perpetrated by a single individual, typically male, through physical force/beating. The victims consisted mostly of women aged between 25 and under 45 years who self-identified as White or mixed race/skin color.

These findings highlight the need for discussion and planning of strategies aimed at preventing and mitigating workplace violence. It is important to emphasize that the notifications and outcomes identified may not fully reflect the reality experienced by workers, given the presence of incomplete information in the records. Therefore, the reporting of workplace violence through SINAN notification forms requires qualified attention from health care teams to ensure more reliable data. This would support the development of future studies and contribute to more effective prevention, protection, and care strategies for workers affected by violence. Furthermore, continuous analysis and dissemination of notification data are essential to allow managers, workers, and other stakeholders to actively engage in surveillance efforts and contribute to the improvement of the system.
